# Estimating Chronic Hepatitis B Prevalence and Undiagnosed Proportion in Canada, 2007-2021: Mathematical Framework Development

**DOI:** 10.2196/66309

**Published:** 2025-08-20

**Authors:** Julien Smith-Roberge, Farinaz Forouzannia, Abdullah Hamadeh, Zeny Feng, Nashira Popovic, William W L Wong

**Affiliations:** 1 School of Pharmacy, University of Waterloo, 10A Victoria St. S., Kitchener, ON, N2G 1C5, Canada, 1 519-888-4567 ext 21323; 2 Department of Mathematics and Statistics, University of Guelph, Guelph, ON, Canada; 3 Centre for Communicable Diseases and Infection Control, Public Health Agency of Canada, Ottawa, ON, Canada

**Keywords:** hepatitis B, prevalence, undiagnosed proportion, mathematical modeling, estimate, Canada, mathematical framework, chronic hepatitis B, CHB, hepatitis, undiagnosed, diagnosis, estimates, prediction, state transition model, liver, liver transplant, hepatocellular carcinoma, decompensated cirrhosis

## Abstract

**Background:**

Chronic hepatitis B (CHB) remains a significant health burden for at least a hundred thousand Canadians. The government of Canada has endorsed the global strategy to eliminate hepatitis as a public health threat by 2030, but effectively targeting public health interventions is complicated by the silent nature of the disease, which can remain asymptomatic for decades.

**Objective:**

This study develops a framework to estimate the prevalence of CHB and the proportion of the infected population that remains undiagnosed. We apply the proposed method to national data from Canada, from 2007 to 2021.

**Methods:**

We infer the prevalence and undiagnosed proportion of CHB by fitting a mathematical state-transition model, based on the natural history of CHB, to observed CHB-related events. Data for the calibration were obtained from the Public Health Agency of Canada and Statistics Canada.

**Results:**

We estimate the national prevalence of CHB in Canada in 2021 to be 0.483% (95% CI 0.443%-0.535%). The corresponding percentage of undiagnosed cases was estimated to be 54.3% (95% CI 50.9%-58.9%).

**Conclusions:**

The estimates of CHB prevalence obtained via our method are in line with previous estimates obtained from national seroprevalence studies. More specialized estimates, stratified by province or age cohort, may be achievable with detailed health administrative data.

## Introduction

Chronic hepatitis B (CHB) is a silent disease. In its early stages, which can last decades, it is largely asymptomatic. Because of this, those with the disease can remain undiagnosed for years until they are alerted to their condition by late-stage complications: decompensated cirrhosis (DC), hepatocellular carcinoma (HCC), and liver failure. It was estimated that hepatitis B caused 1.1 million deaths worldwide in 2022 [1].

In 2016, Canada endorsed the Global Strategy to eliminate hepatitis as a public health threat by 2030 [2]. It has already made progress toward this goal; a seroprevalence study published in 2013 estimated the national prevalence of CHB to be 0.4%, well below the global average [3]. This is in large part thanks to Canada’s robust vaccination program. In 2021, vaccine coverage was 89% for those aged 14 years or older [4]. However, many challenges remain: hepatitis B was one of the most frequently refused vaccines in 2021, third behind only varicella and rotavirus, and the lack of a nationally standardized vaccine schedule means that children who move across provincial lines may miss vaccination due to conflicting schedules [4,5]. Moreover, this vaccination program leaves several key populations underprotected, notably immigrants from countries with high hepatitis B prevalence and children exposed prior to vaccination, a risk that is elevated in provinces that do not have an infant vaccination program. The same 2013 seroprevalence study found that the CHB prevalence among the foreign-born population was 1.6%, 4 times the national average [3], and 90% of unvaccinated infants born to hepatitis B virus (HBV)–infected mothers will develop CHB within 6 months [6]. The risk of contracting CHB also increases with exposure to infected blood and other body fluids, such as through needle sharing or unprotected sex [1].

These at-risk groups comprise a significant portion of Canada’s population. According to the most recent Canadian census published in 2021 [7], immigrants represent 23% of the country’s total population. Many of these originate from regions where HBV is either highly or moderately endemic, notably China and the Philippines, which, along with India, have been the top 3 sources of immigrants to Canada since 1991. As a result, a significant proportion of the CHB population in Canada is comprised of immigrants. Furthermore, Canada’s vaccination program leaves a significant portion of the population underprotected. Many of the provinces still perform adolescent vaccination instead of birth dose vaccination [8]. Other gaps exist in the country’s prevention strategy. For example, in Ontario, prenatal screening for HBV was not universal, with a coverage of 92.7% between 2012 and 2016. These gaps resulted in at least 139 children born between 2003 and 2013 being infected with HBV before being eligible for adolescent vaccination [9].

Meeting Canada’s elimination goals will require further evidence-based interventions, such as screening and community outreach, rooted in robust estimates of CHB prevalence to track the impact and the efficacy of these investments in public health. In this paper, we present a model-based framework for estimating the prevalence and undiagnosed proportion of CHB. Our approach follows in the line of similar back-calculation methods used for other diseases with long incubation periods, such as hepatitis C [10,11] and HIV [12]. Similar methods have also been used to estimate the prevalence and undiagnosed fraction of COVID-19 [13]. To our knowledge, the present study represents the first attempt to apply such back-calculation techniques to CHB.

## Methods

### Overview

In this section, we describe the mathematical framework we use to estimate the prevalence of CHB in populations of interest. We developed a state-transition model of CHB progression based on the natural history of the disease, building on earlier work in [14]. The model includes several late-stage clinical health states, such as DC and HCC, which are more readily diagnosed than the asymptomatic stages of CHB. Indeed, we assume that the symptoms of these late-stage clinical health states are severe enough that they will be diagnosed within 1 year. Using a Bayesian calibration method based on the Metropolis-Hastings algorithm, we used publicly available diagnosis data for these late-stage complications, in combination with diagnosis data for CHB itself, to back-calculate the prevalence and undiagnosed fraction of CHB.

### Natural History Model

**Figure 1.**
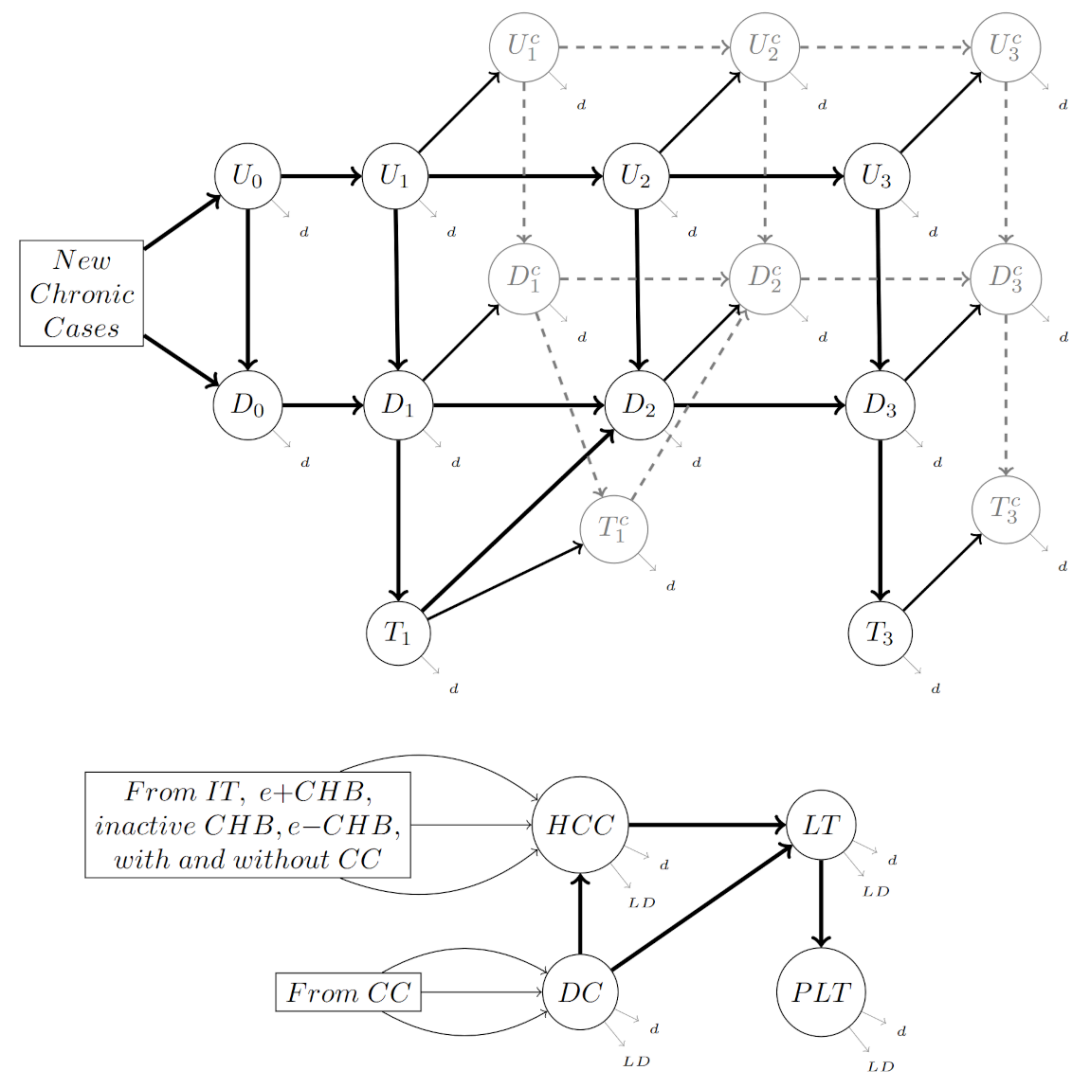
A schematic representation of the state-transition model. CC: compensated cirrhosis; CHB: chronic hepatitis B; DC: decompensated cirrhosis; HCC: hepatocellular carcinoma; LT: liver transplant; PLT: post–liver transplant.

We developed a state transition model to describe the infection, progression, and treatment process, following the natural history of CHB. A schematic of this model is presented in Figure 1. We assume that people infected with CHB will remain in exactly one state for the duration of a given calendar year. Once per year, individuals may transition to a different state based on probabilities derived from the parameters in Table 1 and Multimedia Appendix 1. Some of these state transitions, such as the transition from an undiagnosed state to a diagnosed state, correspond to observable events recorded by public health officials. Our goal is to infer the number of individuals in each state by calibrating the model to reproduce the time evolution of these observables.

**Table 1.** Unknown model parameters. The minimum and maximum constrain the range explored during the calibration process.

Parameter	Description	Min	Max
$d_{l/r}$	Annual probability of CHB^a^ diagnosis	0.005	0.5
$d_{cc}$	Annual probability of CC^b^-specific diagnosis	0	0.1
$d_{aa}$	Probability of newly infected individuals being diagnosed with CHB within the first year	0	0.3
$\mu_{l/r}$	Annual CHB incidence	0	0.1
$r_{hcc0}$	Adjustment to CHB-related HCC^c^ risk	0	1

a CHB: chronic hepatitis B.

b CC: compensated cirrhosis

c HCC: hepatocellular carcinoma.

The structure of our state-transition model is depicted in Figure 1. For clarity, the early stages in the top portion of the diagram are organized along x-, y-, and z-axes based on their nature. The states are organized in rows and denoted by U, D, and T, corresponding to the cascade of care, specifically, the undiagnosed and untreated states, for those who have CHB but are unaware of their infection; the diagnosed and untreated states, for those with a positive CHB diagnosis who have not initiated treatment; and diagnosed and treated states, for those receiving treatment for their CHB infection. Each of these basic states is further subdivided into sub-stages, organized into columns, that represent the natural history of CHB. These sub-stages are denoted using a subscript. The subscript 1 denotes chronic hepatitis B e-antigen (HBeAg) + hepatitis B (hepatitis B surface antigen [HBsAg]: positive, HBeAg: positive, viral load: high, ALT level: high). The subscript 2 denotes Inactive Hepatitis B (HBsAg: positive, HBeAg: negative, viral load: low, ALT level: normal). The subscript 3 denotes Chronic HBeAg-Hepatitis B (HBsAg: positive, HBeAg: negative, viral load: high, ALT level: high).

Finally, each of these states can exist with or without compensated cirrhosis (CC), which, when present, is represented with a superscript C. In order to cover the whole natural history of CHB, we also include 2 states, $U_{0}$ and $D_{0}$, corresponding to the immune tolerant state (HBsAg: positive, HBeAg: positive, viral load: high, ALT level: normal).

People infected with CHB may progress into advanced stages of liver disease, which are depicted in the bottom half of Figure 1. These advanced stages are categorized into the following states: HCC, DC, liver transplant (LT), and post–liver transplant (PLT).

We define the LT state to last at most 1 year. Transplant recipients who have survived more than 1 year after their liver transplantation enter the PLT state. Additionally, we assume that individuals in these later states have a probability of mortality greater than the background all-cause mortality. This is represented by arrows to a liver death state, LD. Background mortality is represented by an arrow to a death state, d. A full list of model states is included in Multimedia Appendix 2.

Finally, we assume that in the year *t,* there are u(t) new CHB infections. These new infections include observed infections that are diagnosed within the first year, in which case they will be directed to the state $D_{0}$, as well as unobserved cases that remain undiagnosed and go to $U_{0}$.

### Model Transition Probabilities

The transition probabilities of the model depend on a mix of known and unknown parameters. The known parameters are obtained from the literature and are presented in Multimedia Appendix 1. The unknown parameters are presented in (Table 1). We will estimate these unknown parameters using our calibration procedure, which is described in later sections of this paper.

Because CHB is initially asymptomatic, we assume that the probability of receiving a diagnosis is constant across all the undiagnosed states *U*_*i*_. In principle, these diagnosis probabilities can vary significantly from year to year. As a simplifying approximation, we assume that this trend is linear over the period being modeled, with a diagnosis probability of $d_{l}$ in the first year being modeled, $t_{init}$, and a final diagnosis probability of $d_{r}$ in the last year modeled, $t_{fin}$. For notational convenience, we denote this time-dependent quantity $d_{l/r}(t)$. For undiagnosed states with compensated cirrhosis, we add an annual probability of CC-specific diagnosis, $d_{cc}$, to allow for the fact that compensated cirrhosis may likely produce symptoms, which increases the probability of diagnosis. Thus, the diagnosis probability for an individual in one of the $U_{i}^{C}$ states would be $d_{l/r}\left(t\right)+d_{cc}$. Finally, we apply a different diagnosis probability for the first year of infection, $d_{aa}$. In any given year, $d_{aa}u(t)$ new cases will be added to the $D_{0}$ state, with the remaining $\left(1-d_{aa}\right)u\left(t\right)$ cases being added to the $U_{0}$ state. This first-year diagnosis probability allows us to account for CHB-infected individuals who seek screening following high-risk behavior or immigrants who disclose their health status on entering the country.

Much like the diagnosis probability, we also assume that CHB incidence, μ, is a linear function of time. Following the same notational convention as earlier, the number of new cases in a given year is given by $u(t)=\mu_{l/r}(t)\cdot \text{Pop}(t)$, where Pop(t) is the population of the cohort being studied. We note that several of the parameter extrema, such as a CHB incidence of 0.1, are set implausibly high to grant the model the flexibility to explore all plausible parameter values.

The final calibration parameter, $r_{hcc0}$, is an adjustment parameter that aims to account for uncertainty in the probability of developing HCC. The annual probabilities of developing HCC, as reported in the literature, are compiled in Multimedia Appendix 1.

Finally, we note that these model parameters, both known and unknown, represent the underlying probabilities associated with any basic state transition. In principle, simulating the full state transition model represented by Figure 1 would also require knowledge of joint probabilities. In order to obtain a tractable model, we make the simplifying assumption that each of these basic events is independent.

### Model Assumptions

We highlight some key simplifying assumptions made during the development of the model (Textbox 1).

Textbox 1. **Model assumptions.**

- Incidence rate is a linear function of time and does not explicitly depend on other factors, such as vaccine uptake.
- The age distribution of the population with chronic hepatitis B (CHB) matches that of the general population. In particular, age-related quantities, such as the background mortality rate, are the same in both populations.
- There is no net migration of infected individuals after arrival. Moreover, because CHB has no readily available cure (spontaneous loss of hepatitis B surface antigen [HBsAg] is possible, but rare), we assume that the number of cases can only decrease by death.
- Treatment uptake and efficacy remained constant over the observed period, as did survival rates of late-stage complications.

### Model Dynamics

For each of the model states ($U_{0}$, $D_{0}$, $U_{1}$, ..., HCC, DC, LT, and PLT), the state-transition model generates a time series for the expected number of individuals in each state. We denote these by $U_{0}(t)$, $D_{0}(t)$, ..., and PLT(t), for $t=t_{init},...,t_{fin}$. Additionally, we record the number of individuals transitioning between states. These simulated time series—the number of individuals in each state and the number of individuals transitioning between states—are used to calculate 4 *observables*, which are used to calibrate the model. The first of these is the number of people receiving treatment, $T_{1}\left(t\right)+T_{3}\left(t\right)+T_{1}^{C}\left(t\right)+T_{3}^{C}\left(t\right)$. Based on clinical guidelines [15], treatment is mainly offered to infected individuals in HBeAg+ and HBeAg- states, and thus our model does not include an inactive treatment state, $T_{2}$. The remaining observables are the number of CHB diagnoses, the number of HCC diagnoses, and the number of late HCC diagnoses, which we define as any transition from an undiagnosed state, $U_{i}^{-/C}$, to the HCC state. These diagnosis events correspond to transitions between undiagnosed states to diagnosed states. The details of these computations can be found in (Multimedia Appendix 2).

### Data Sources

The observables described earlier are compared to 3 data sources: the annual number of HCC diagnoses, obtained from Statistics Canada [16], which we will denote$\;Y_{HCCTotal}\left(t\right)$; the annual number of CHB diagnoses, obtained from the Public Health Agency of Canada’s (PHAC) Notifiable Diseases Online [17], denoted $Y_{CHB}(t)$; and CHB treatment data, which is based on prescription data obtained from IQVIA via collaboration with PHAC, denoted $Y_{Treat}\left(t\right)$. The CHB and treatment data can be directly compared to their corresponding observables, but the other 2 observables require additional assumptions on the data before they can be used.

The HCC figures from Statistics Canada include all instances of HCC, regardless of cause. Following [14], the estimated number of CHB-related HCC cases is 9% of total HCC cases,$\;Y_{HCC}\left(t\right)\;=\;0.09\cdot Y_{HCCTotal}\left(t\right)$.

In [18], an estimated 1.07% of CHB diagnoses (198 of 18,434) occur within ±6 months of an HCC diagnosis. Thus, we assume the number of late HCC diagnoses per year is given by

.$$Y_{LateHCC}(t)=0.0107\cdot Y_{CHB}(t)$$

Reporting HBV cases is mandatory. Therefore, we can be confident that the Notifiable Diseases Online database accurately reflects the number of HBV diagnoses. However, although HBV diagnoses must be reported, the chronic versus acute distinction is not reportable in all provinces and territories. Unspecified cases were apportioned based on the chronic-to-acute ratio observed in the specified cases. We note that although the rate of HBV diagnosis per se may be biased (not all people who are infected will seek medical attention, and therefore the Notifiable Diseases database might systematically underrepresent populations less likely to interact with the health care system), the structure of our mathematical model accounts for this possibility. Bias or underreporting in the diagnoses of late-stage complications represents a greater source of uncertainty in the model. As such, the HCC data from Statistics Canada represents the most significant source of data-related uncertainty in our analysis. The data for model calibration are available in Multimedia Appendix 3.

### Model Calibration

The model contains several unknown quantities that need to be inferred via model calibration. These come in 2 broad categories: the unknown parameters from Table 1, and the initial population sizes for each of the model states.

If the initial population distributions in the year $t_{init}$ deviate too much from the true values, the calibration converges to a spurious local optimum. In order to avoid this, we use a 2-stage calibration process. We begin by assuming a prevalence of 0.4%, in line with earlier seroprevalence results [3], and distribute these among the states in the model based on percentages used in previous modeling studies [19]. We then calibrated the model against data from a subset of provinces—Alberta, British Columbia, Ontario, and Saskatchewan—whose diagnosis, treatment, and HCC data were the most complete and selected the population distribution that yielded the closest match to their data. These population distributions then served as our initial distribution in all subsequent calibrations.

In order to obtain an initial estimate of incidence, $\mu_{l/r}$, we assume the system is in approximate equilibrium and apply the same 0.4% seroprevalence estimate. This yields a preliminary estimate of $\mu_{l/r}\approx 0.00004$.

In principle, the early diagnosis parameter, $d_{aa}$, can take any value between 0 and 1, though in practice we know that values close to 1 are unrealistic. We restrict our search range to $0\leq d_{aa}\leq 0.3$ [18].

### Calibration Algorithm

Following [10], we use a 2-stage calibration algorithm, which is designed to account for the uncertainty in the literature-derived parameters. Let $v^{lit}=[q_{01},...,d_{ld4}]$ be a set of the literature-derived model parameters, ie, a set of parameters sampled from the ranges in Multimedia Appendix 1. Similarly, let $v^{cal}=\;\left[d_{l},...,r_{hcc0},U_{0}\left(t_{init}\right),...,PLT\left(t_{init}\right)\right]$ denote a set of the calibration parameters, ie, a set of the unknown model parameters from Table 1 and a set of initial population estimates. Taken together, a single pair $(v^{lit},v^{cal})$ determines the initial conditions and transition probabilities, and thus fully specifies the behavior of the state transition model. The goal of our calibration is to generate pairs that yield a good fit with the available data.

Our 2-stage calibration algorithm is described as follows:

*Stage 1:* We begin by generating k sets of the literature-derived parameters,

$\left[v_{1}^{lit},\ldots ,v_{k}^{lit}\right]$, by sampling uniformly from the ranges in Multimedia Appendix 1. For each sample $v_{i}^{lit}$, we calibrate the model using the Metropolis-Hastings MCMC algorithm [20], using the likelihood function described in Multimedia Appendix 2, until the Markov chain converges to a stationary distribution, $P\left(v^{cal}|Y,v_{i}^{lit}\right)$. Discarding the burn-in, we obtain a set of n samples for the unknown parameters, $\left[v_{1}^{cal},\ldots ,v_{n}^{cal}\right]$, sampled from this distribution.

*Stage 2:* The samples generated in the first stage are conditioned on a choice of $v_{i}^{lit}$. Thus, in order to obtain a full set of model parameters, we perform a second sampling step. We first sample uniformly from the literature-derived parameters obtained in step 1, $\left[v_{1}^{lit},\ldots ,v_{k}^{lit}\right]$, and then sample uniformly from the associated distribution obtained via the MCMC algorithm. The result is a single combined set of parameters, $\left(v^{lit},v^{cal}\right)$, sampled from the distribution $P\left(v^{lit},v^{cal}|Y\right)$.

For each pair $\left(v^{lit},v^{cal}\right)$ derived in this way, the model produces a time series for each of the model states, $U_{0}(t)$, $D_{0}\left(t\right)$, ..., PLT(t). In order to describe the typical behavior of our model, we sample multiple pairs $\left(v^{lit},v^{cal}\right)$ and compute the means of the associated time series, $\bar{U_{0}}\left(t\right)$, $\bar{D_{0}}\left(t\right)$, ..., $\bar{PLT}\left(t\right)$, as well as the corresponding standard deviations.

We obtain a prevalence estimate by summing over all model states and dividing by the total population size.


$$CHB\;prevalence(t)=[{\bar{U}}_{0}(t)+{\bar{D}}_{0}(t)+...+P\bar{L}T(t)]/Pop(t)$$

The undiagnosed fraction is computed as the ratio of all undiagnosed states and the total CHB.

U


$$Undiagnosed\;fraction(t)=\frac{{\bar{U}}_{0}(t)+{\bar{U}}_{1}(t)+...+{\bar{U}}_{3}^{C}(t)}{{\bar{U}}_{0}(t)+{\bar{D}}_{0}(t)+...+P\bar{L}T(t)}$$

Using this 2-stage calibration algorithm, we first estimate the national prevalence. We then conduct a stratified analysis for male and female populations. Finally, we conduct a one-way sensitivity analysis to explore the uncertainty associated with these estimates.

### Ethical Considerations

This study uses publicly deidentified data collected by Public Health Agency of Canada and Statistics Canada. The analysis procedure has been approved by the Research Board at the University of Waterloo (#43995).

## Results

**Figure 2.**
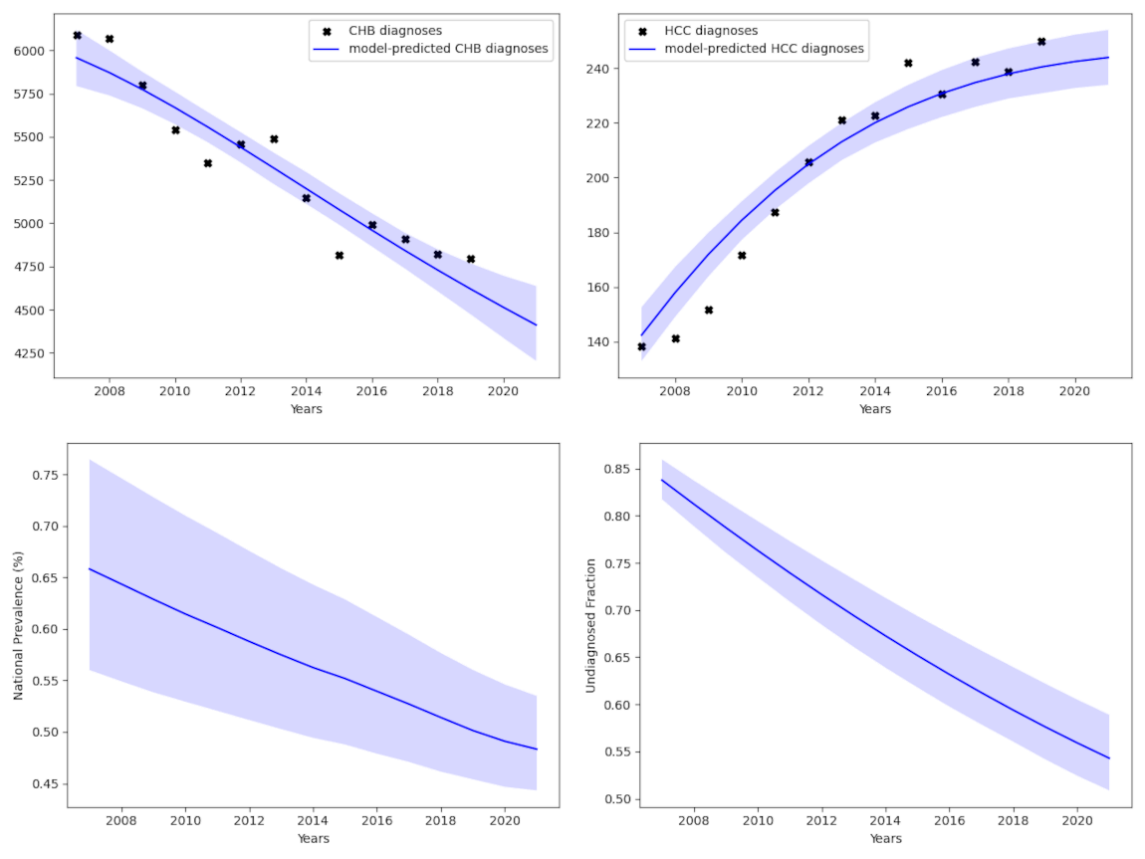
Estimates of the national chronic hepatitis B (CHB) prevalence across all cohorts. The top 2 plots show the model fit compared to data for CHB diagnoses (top left) and CHB-attributed hepatocellular carcinoma (HCC) diagnoses (top right). The bottom 2 plots accordingly display the estimates of prevalence (bottom left) and of the undiagnosed proportion (bottom right).

After completing the calibration process outlined earlier, the algorithm identified a set of model parameters and initial conditions that can closely match with the observed statistics on CHB diagnosis and HCC diagnosis (Figure 2). In order to produce such a fit, the national prevalence of CHB in 2021 was estimated to be 0.483% (95% CI 0.443%‐0.535%), while the proportion undiagnosed in 2021 was estimated to be 54.3% (95% CI 50.9%‐58.9%; Figure 2). The model had a deviance information criterion of 53.9.

### Stratified Analyses

**Figure 3.**
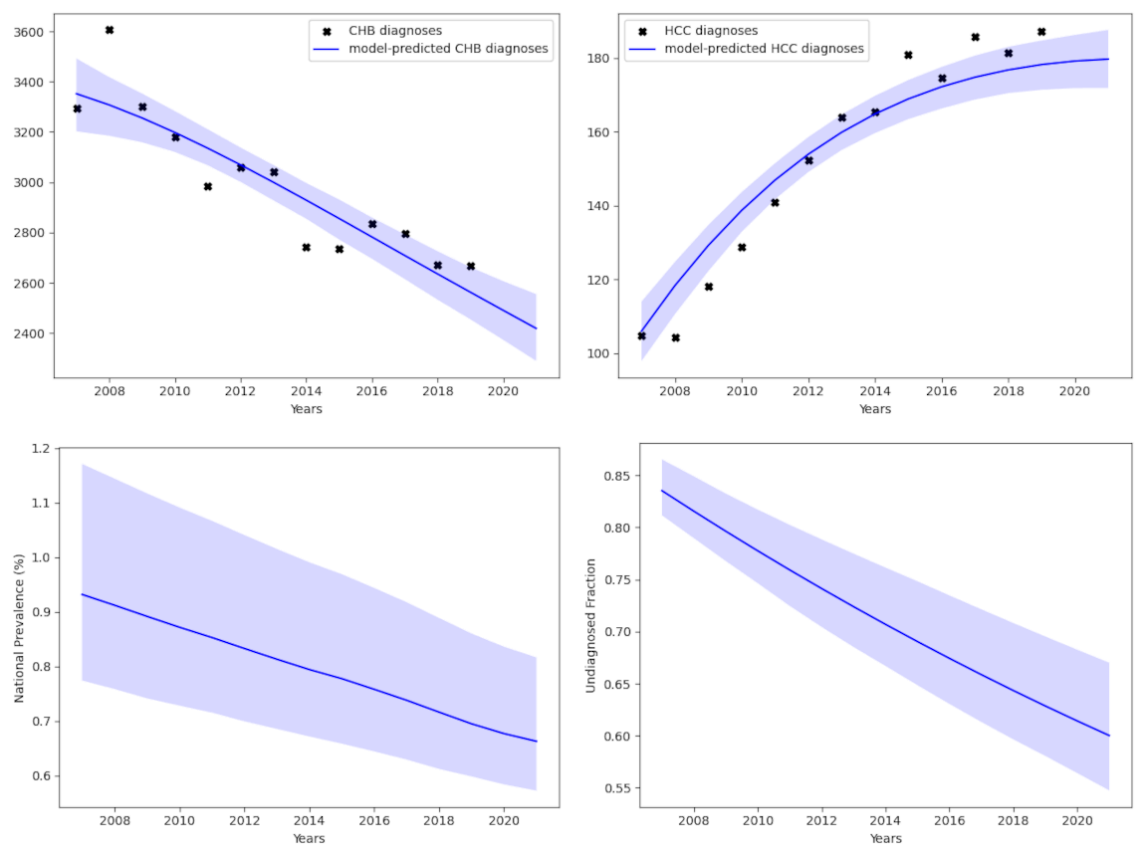
Estimates of the chronic hepatitis B (CHB) prevalence in males. The top two plots show the model fit compared to data for CHB diagnoses (top left) and CHB-attributed hepatocellular carcinoma (HCC) diagnoses (top right). The bottom 2 plots accordingly display the estimates of prevalence (bottom left) and of the undiagnosed proportion (bottom right).

**Figure 4.**
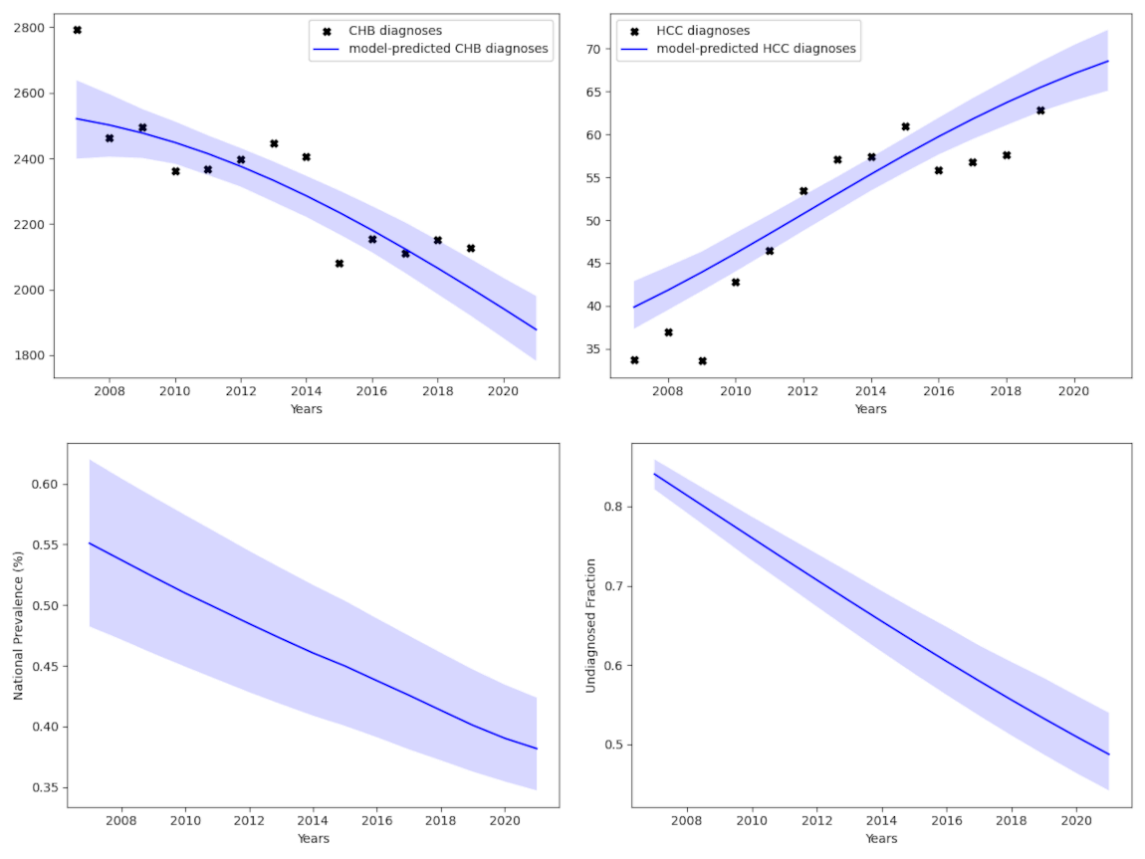
Estimates of the chronic hepatitis B (CHB) prevalence in females. The top two plots show the model fit compared to data for CHB diagnoses (top left) and CHB-attributed hepatocellular carcinoma (HCC) diagnoses (top right). The bottom 2 plots accordingly display the estimates of prevalence (bottom left) and of the undiagnosed proportion (bottom right).

We conducted a second calibration stratified by sex. Again, the algorithm identified a set of model parameters and initial conditions that closely match the observed statistics on CHB diagnosis and HCC diagnosis for the male population (Figure 3) and the female population (Figure 4). In order to produce these fits, the stratified analyses suggest a slightly higher prevalence of 0.663% (95% CI 0.572%‐0.816%) in the male population, while the proportion undiagnosed in 2021 was estimated to be 60.0% (95% CI 54.8%‐67.0%; Figure 3). In the female population, we obtain a slightly lower prevalence of 0.382% (95% CI 0.347%‐0.424%), while the proportion undiagnosed in 2021 was estimated to be 48.8% (95% CI 44.2%‐54.0%; Figure 4). The male and female fits had deviance information criteria of 9.0 and 73.2, respectively. The differences in prevalence obtained from this calibration process are consistent with the known sexual dimorphism in CHB [21].

In addition to stratifying by sex, we also attempted an analysis based on age cohort. However, because late-stage complications are highly skewed toward older individuals, the dataset for the younger cohort was too small and noisy to make reliable inferences. We intend to address this in future work with better health administrative data.

### Sensitivity Analysis

**Figure 5.**
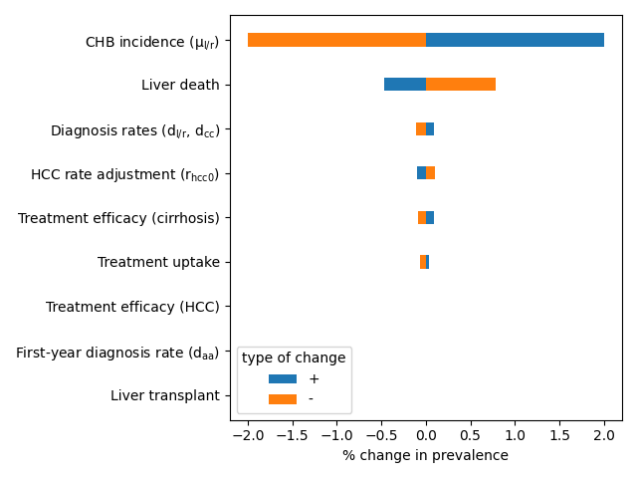
One-way sensitivity analysis for the estimated prevalence of chronic hepatitis B (CHB). HCC: hepatocellular carcinoma.

We performed a 1-way sensitivity analysis of the calibrated model, varying the parameters in Table 1 to determine which components of the model caused the greatest variation in the estimates of the prevalence and undiagnosed fraction. Parameters were varied by ±50%, and we measured the resulting change in the prevalence and diagnosed fraction, averaged over the time period studied (2007‐2021). The results are presented in Figures5  6.

**Figure 6.**
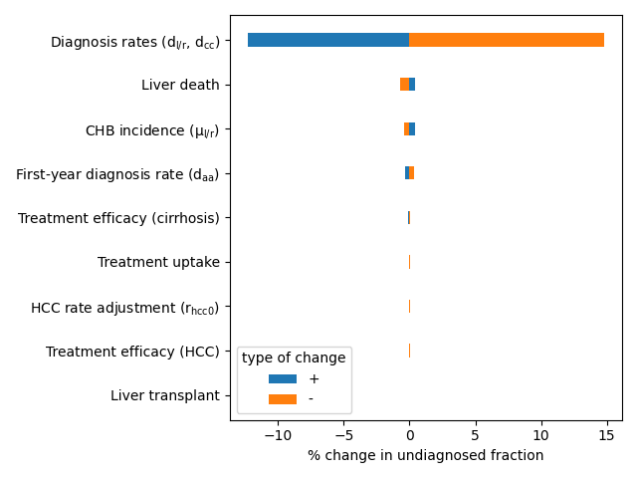
One-way sensitivity analysis for the estimated undiagnosed proportion of CHB infections. CHB: chronic hepatitis B; HCC: hepatocellular carcinoma.

As expected, the yearly CHB incidence has the largest impact on prevalence. Other parameter changes impact prevalence indirectly by changing the mortality rate. This effect was seen most strongly in the probability of liver death associated with late-stage complications. Changing the diagnosis probabilities or HCC incidence also increased prevalence, presumably because of their downstream effects on CHB-related mortality. Similarly, increasing the treatment uptake and treatment effectiveness increased prevalence due to the associated drop in mortality. The effects of $d_{aa}$ on prevalence were negligible.

Changes in the diagnosis probabilities had the largest effect on the undiagnosed fraction, followed by the probability of liver death, CHB incidence, and $d_{aa}$, which had comparable effects. Changes in events downstream of diagnosis, such as HCC incidence or treatment effectiveness, did not change the overall prevalence enough to have a significant impact on the undiagnosed proportion.

## Discussion

### Principal Results

CHB has a long incubation period, often progressing undetected until the onset of late-stage complications. This creates significant uncertainties in the prevalence and undiagnosed proportion and poses challenges to policymakers who wish to measure the efficacy of public health interventions or track progress toward elimination targets. We have proposed a model-based method to estimate these quantities. Using limited CHB and HCC diagnosis data, we estimate prevalence in 2021 to be 0.483% (95% CI 0.443%‐0.535%). These results are in line with previous 0.4% seroprevalence estimates [3]. Additionally, the proposed method provides estimates of the fraction of CHB cases that remain undiagnosed, in this case 54.3% (95% CI 50.9%‐58.9%). Again, this is in line with previous estimates. The same Canadian seroprevalence study indicated that 46% of respondents who tested positive for a present HBV infection knew of their infected status.

These numbers are comparable to estimates obtained in other countries. In 2022, approximately 205,549 individuals in Australia were living with CHB, representing 0.78% of the population. It is estimated that 27.9% of these remain undiagnosed [22]. The prevalence of hepatitis B varies across European Union/European Economic Area countries, with estimates ranging from 0.1% in Ireland to 4.4% in Romania [23]. The proportion of undiagnosed infections also varies widely, ranging between 45% and 85%, highlighting gaps in national testing programs [24]. Although our results are focused on Canada, other high-income, immigrant-receiving countries like the United Kingdom and Australia may also benefit from applying the methods developed in this paper.

A 1-way sensitivity analysis identifies CHB incidence as the greatest factor influencing overall prevalence, followed by the probability of liver death in late-stage clinical health states, followed by treatment efficacy against cirrhosis. The undiagnosed fraction was sensitive to changes in diagnosis probabilities and CHB incidence, but treatment efficacy did not impact prevalence enough to have a significant effect on the undiagnosed proportion.

### Limitations

The estimation method we have presented has several advantages. For one, it is systematic: the analysis we have performed can, in principle, be extended to any cohort for which data are available. Additionally, the 2-step calibration method accounts for uncertainty in the model parameters that are derived from the literature, yielding statistically robust estimates. However, a few important limitations constrain its use. We made the simplifying assumption that the CHB incidence is a linear function of time, but the 1-way sensitivity analysis identified CHB incidence as the parameter with the largest impact on prevalence. Therefore, the assumption of linearity greatly limits the time resolution of the model. As such, the time series presented in this paper should be taken as indicators of overall prevalence trends, rather than a detailed year-by-year accounting. In particular, the model incidence as currently formulated cannot accommodate sudden changes in vaccine uptake or immigration rates. This latter limitation may be addressed by immigrant sub-cohort modeling, though we currently lack the data required to perform this analysis. There is also considerable uncertainty surrounding HCC incidence. For one, the HCC data we use for calibration include HCC from all causes. Following [14], we estimate that hepatitis B accounts for 9% of HCC cases, but this estimate does not account for annual variation. Moreover, as with all cancers, HCC incidence is known to be highly age-dependent, but the available HCC data were not detailed enough to calibrate a state transition model that accounts for age. This latter limitation makes it impossible to reliably stratify our analysis by age cohort. Fortunately, both of these HCC-related limitations can be addressed with more accurate health administration data.

### Conclusions

With these limitations in mind, our results point to some notable public health concerns. Despite Canada’s 2016 endorsement of ambitious elimination targets, our 2021 CHB prevalence estimate does not indicate a significant decrease over previous estimates from 2013. Hepatitis B continues to be a substantial health burden for at least a hundred thousand Canadians. Of these, a significant proportion remains undiagnosed and untreated, putting them at increased risk of serious complications. Our analysis suggests that measures to decrease CHB incidence, such as vaccination and screening, have the greatest potential to decrease the overall disease burden. Based on our current data limitations, further stratified analysis is not possible. Our next step will be accessing health administrative data to overcome this barrier, with the aim of producing more reliable and detailed estimates.

We have presented a method to estimate the prevalence of CHB, using a state transition model based on the natural history of the disease. The estimates are obtained via a 2-stage calibration process that incorporates multiple sources of uncertainty, yielding results that are robust to perturbations of the model. This calibration process relies only on diagnosis data, which are already collected by the health care system, making it an attractive alternative to seroprevalence studies and other methods that require the collection of novel data. Conversely, a lack of high-quality health administrative data significantly limits the accuracy of the model. This is especially true among small populations and younger age cohorts, as younger individuals will typically not have developed a large and statistically robust pattern of late-stage complications. With more data, these estimates can further be stratified by region or by age cohort, allowing them to inform the targeting of public health interventions.

## Supplementary material

10.2196/66309 Multimedia Appendix 1 Literature-derived model parameters.

10.2196/66309 Multimedia Appendix 2 Model dynamics and likelihood.

10.2196/66309 Multimedia Appendix 3 Calibration data.

## Abbreviations

CC: compensated cirrhosis
CHB: chronic hepatitis B
DC: decompensated cirrhosis
HBeAg: hepatitis B e-antigen
HBsAg: hepatitis B surface antigen
HBV: hepatitis B virus
HCC: hepatocellular carcinoma
LT: liver transplant
PHAC: Public Health Agency of Canada
PLT: post–liver transplant
