# Supplementary material for: Estimating Chronic Hepatitis B Prevalence and Undiagnosed Proportion in Canada, 2007-2021: Mathematical Framework Development
Source: JMIR Public Health Surveill. 2025 Aug 20;11:e66309. doi: 10.2196/66309 (PMC12367349; doi:10.2196/66309)
Supplement: Multimedia Appendix 1 [file publichealth-v11-e66309-s001.docx]

# Appendix 1: Literature-derived Model Parameters

Table 1: Transition probabilities used in the state-transition model. Parameter values are sampled uniformly from the range dictated by the minimum and maximum values.

| Parameter | Annual probability that... | Min | Max | Source |
| --- | --- | --- | --- | --- |
| *q*01 | an untreated individual in the IT state will progress to the e+CHB state | 0.12 | 0.16 | [1] |
| *q*12 | an untreated individual in the e+CHB state will progress to the inactive CHB state | 0.0079 | 0.0551 | [2] [3] |
| *q*23 | an untreated individual in the inactive CHB state will progress to the e-CHB state | 0.02 | 0.05 | [4] [5] [6] |
| *dcc*1 | an untreated individual in the e+CHB state will progress to the CC state | 0.022 | 0.088 | [4] [7] [8] [9] |
| *dcc*2 | an untreated individual in the inactive CHB state will progress to the CC state | 0.001 | 0.002 | [4] [5] [7] |
| *dcc*3 | an untreated individual in the e-CHB state will progress to the CC state | 0.015 | 0.058 | [4] [5] [10] [11] |
| *t_i_* | an untreated individual will begin treatment | 0.1775 | 0.2958 | [12] |
| *dhcc*0 | an untreated individual in the IT state will develop HCC | 0 | 0.0004 | [13] |
| *dhcc*1 | an untreated individual in the e+CHB state will develop  HCC | 0.004 | 0.016 | [7] [14] [15] |
| *dhcc*2 | an untreated individual in the inactive CHB state will develop HCC | 0.0015 | 0.006 | [5] [7] |
| *dhcc*3 | an untreated individual in the e-CHB state will develop  HCC | 0.004 | 0.012 | [5] [13] [15] [16] |
| *dhcc*1*cc* | an untreated individual in the e+CHB state (with CC) will develop HCC | 0.01 | 0.12 | [4] [17] [18] [19] |
| *dhcc*2*cc* | an untreated individual in the Inactive state (with CC) will develop HCC | 0.011 | 0.044 | [4] [7] [20] |
| *dhcc*3*cc* | an untreated individual in the e-CHB state (with CC) will develop HCC | 0.01 | 0.12 | [4] [15] [17] [18] [19] |
| *dthcc*1 | a treated individual in the e+CHB state will develop HCC | 0.0023 | 0.0038 | [21] + untreated # |
| *dthcc*3 | a treated individual in the e-CHB state will develop HCC | 0.0023 | 0.0038 | [22] + untreated # |
| *dthcc*4 | a treated individual in the CC state will develop HCC | 0.0098 | 0.0164 | [22] [23] + untreated # |
| *dDCC*1 | an untreated individual in the e+CHB state (with CC) will develop DC | 0.035 | 0.1 | [4] [19] [24] [25] |
| *dDCC*2 | an untreated individual in the Inactive state (with CC) will develop DC | 0.004 | 0.016 | [4] [7] [20] |
| *dDCC*3 | an untreated individual in the e-CHB state (with CC) will develop DC | 0.035 | 0.1 | [4] [19] [24] [25] |
| *dtDCC*4 | a treated individual in the CC state will develop DC | 0.0285 | 0.0475 | [21] + untreated # |
| *dtcc*1 | a treated individual in the e+CHB state will progress to the  CC state | 0.0102 | 0.017 | [21] + untreated # |
| *qt*12 | a treated individual in the e+CHB state will progress to the inactive CHB state | 0.0434 | 0.1422 | [2] [3] |
| *qt*34 | a treated individual in the e-CHB state will progress to the  CC state | 0.0067 | 0.0111 | [21] + untreated # |
| *dhccdc* | an individual in the DC state will transit to state HCC | 0.01 | 0.113 | [4] [15] [26] [27] |
| *dhcclt* | an individual in the HCC state will get a liver transplant | 0.05 | 0.4 | [4] [26] [27] |
| *ddclt* | an individual in the DC state will get a liver transplant | 0 | 0.4 | [4] [26] [27] |
| *dld*1 | an individual in the DC state will suffer liver death | 0.058 | 0.221 | [4] [26] [27] |
| *dld*2 | an individual in the HCC state will suffer liver death | 0.181 | 0.451 | [4] [26] [27] |
| *dld*3 | an individual will suffer liver death the same year they receive a liver transplant | 0.124 | 0.159 | [28] |
| *dld*4 | an individual will suffer liver death following a liver transplant in the subsequent treatment | 0.024 | 0.043 | [28] |
|  | RR of CHB to CC for treatment vs no treatment | 0.231 | 0.385 | [21] |
|  | RR of CC to DC for treatment vs no treatment | 0.3910 | 0.6510 | [21] |
|  | RR of CC to HCC for treatment vs no treatment | 0.2892 | 0.4821 | [22] [23] |
|  | RR of CHB to HCC for treatment vs no treatment | 0.15 | 0.91 | [22] |

# References

1. Chu CM, Liaw YF. Chronic hepatitis B virus infection acquired in childhood: special emphasis on prognostic and therapeutic implication of delayed HBeAg seroconversion. Journal of viral hepatitis. 2007;14(3):147-52.
2. Chan HL, Fung S, Seto WK, Chuang WL, Chen CY, Kim HJ, et al. Tenofovir alafenamide versus tenofovir disoproxil fumarate for the treatment of HBeAg-positive chronic hepatitis B virus infection: a randomised, doubleblind, phase 3, non-inferiority trial. The lancet Gastroenterology & hepatology. 2016;1(3):185-95.
3. Wong WW, Pechivanoglou P, Wong J, Bielecki JM, Haines A, Erman A, et al. Antiviral treatment for treatment-na¨ıve chronic hepatitis B: systematic review and network meta-analysis of randomized controlled trials. Systematic Reviews. 2019;8:1-15.
4. Wong WW, Woo G, Jenny Heathcote E, Krahn M. Cost effectiveness of screening immigrants for hepatitis B. Liver International. 2011;31(8):117990.
5. Hsu YS, Chien RN, Yeh CT, Sheen IS, Chiou HY, Chu CM, et al. Longterm outcome after spontaneous HBeAg seroconversion in patients with chronic hepatitis B. Hepatology. 2002;35(6):1522-7.
6. Gigi E, Lalla T, Orphanou E, Sinakos E, Vrettou E, Raptopoulou-Gigi M. Long term follow-up of a large cohort of inactive HBsAg (+)/HBeAg ()/anti-HBe (+) carriers in Greece. Journal of Gastrointestinal and Liver Diseases: JGLD. 2007;16(1):19-22.
7. Veenstra DL, Sullivan SD, Clarke L, Iloeje UH, Tafesse E, Di Bisceglie A, et al. Cost effectiveness of entecavir versus lamivudine with adefovir salvage in HBeAg-positive chronic hepatitis B. Pharmacoeconomics. 2007;25:96377.
8. Liaw YF, Tai DI, Chu CM, Chen TJ. The development of cirrhosis in patients with chronic type B hepatitis: a prospective study. Hepatology. 1988;8(3):493-6.
9. Lin SM, Sheen IS, Chien RN, Chu CM, Liaw YF. Long-term beneficial effect of interferon therapy in patients with chronic hepatitis B virus infection. Hepatology. 1999;29(3):971-5.
10. Iloeje UH, Yang HI, Su J, Jen CL, You SL, Chen CJ, et al. Predicting cirrhosis risk based on the level of circulating hepatitis B viral load. Gastroenterology. 2006;130(3):678-86.
11. Veenstra D, Spackman D, Bisceglie A, Kowdley K, Gish R. Evaluating anti-viral drug selection and treatment duration in HBeAg-negative chronic hepatitis B: a cost-effectiveness analysis. Alimentary pharmacology & therapeutics. 2008;27(12):1240-52.
12. Razavi-Shearer D, Gamkrelidze I, Nguyen MH, Chen DS, Van Damme P, Abbas Z, et al. Global prevalence, treatment, and prevention of hepatitis B virus infection in 2016: a modelling study. The lancet Gastroenterology & hepatology. 2018;3(6):383-403.
13. Wen WH, Chang MH, Hsu HY, Ni YH, Chen HL. The development of hepatocellular carcinoma among prospectively followed children with chronic hepatitis B virus infection. The Journal of pediatrics. 2004;144(3):397-9.
14. Liaw YF, Tai DI, Chu CM, Lin DY, Sheen IS, Chen TJ, et al. Early detection of hepatocellular carcinoma in patients with chronic type B hepatitis: a prospective study. Gastroenterology. 1986;90(2):263-7.
15. Papatheodoridis GV, Manolakopoulos S, Touloumi G, Vourli G, Raptopoulou-Gigi M, Vafiadis-Zouboulis I, et al. Risk of hepatocellular carcinoma (HCC) in chronic hepatitis b (CHB) patients with or without cirrhosis treated with oral antivirals: results of the nationwide hepnet. Greece cohort study: 137. Hepatology. 2009;50:368A-369A.
16. Chen CJ, Yang HI, Su J, Jen CL, You SL, Lu SN, et al. Risk of hepatocellular carcinoma across a biological gradient of serum hepatitis B virus DNA level. Jama. 2006;295(1):65-73.
17. Benvegnu L, Gios M, Boccato S, Alberti A. Natural history of compensated viral cirrhosis: a prospective study on the incidence and hierarchy of major complications. Gut. 2004;53(5):744-9.
18. Fattovich G, Giustina G, Schalm SW, Hadziyannis S, Sanchez-Tapias J, Almasio P, et al. Occurrence of hepatocellular carcinoma and decompensation in western European patients with cirrhosis type B. Hepatology. 1995;21(1):77-82.
19. Kanwal F, Farid M, Martin P, Chen G, Gralnek IM, Dulai GS, et al. Treatment alternatives for hepatitis B cirrhosis: a cost-effectiveness analysis. Official journal of the American College of Gastroenterology— ACG. 2006;101(9):2076-89.
20. Fattovich G, Pantalena M, Zagni I, Realdi G, Schalm SW, Christensen E, et al. Effect of hepatitis B and C virus infections on the natural history of compensated cirrhosis: a cohort study of 297 patients. The American journal of gastroenterology. 2002;97(11):2886-95.
21. Dakin H, Bentley A, Dusheiko G. Cost–utility analysis of tenofovir disoproxil fumarate in the treatment of chronic hepatitis B. Value in Health. 2010;13(8):922-33.
22. Hosaka T, Suzuki F, Kobayashi M, Seko Y, Kawamura Y, Sezaki H, et al. Long-term entecavir treatment reduces hepatocellular carcinoma incidence in patients with hepatitis B virus infection. Hepatology. 2013;58(1):98-107.
23. Wong GLH, Chan HLY, Mak CWH, Lee SKY, Ip ZMY, Lam ATH, et al. Entecavir treatment reduces hepatic events and deaths in chronic hepatitis B patients with liver cirrhosis. Hepatology. 2013;58(5):1537-47.
24. Craxi A, Colombo P, D’Amico G, Di Blasi F, Di Marco V, Magrin S, et al. Hepatitis B infection and liver cirrhosis: a reappraisal from the Mediterranean area. Annali Dell’istituto Superiore di Sanita. 1987;24(2):257-65.
25. D’amico G, Morabito A, Pagliaro L, Marubini E, of “V Cervello” Hospital LSG. Survival and prognostic indicators in compensated and decompensated cirrhosis. Digestive diseases and sciences. 1986;31:468-75.
26. Hutton DW, Tan D, So SK, Brandeau ML. Cost-effectiveness of screening and vaccinating Asian and Pacific Islander adults for hepatitis B. Annals of internal medicine. 2007;147(7):460-9.
27. Kanwal F, Gralnek IM, Martin P, Dulai GS, Farid M, Spiegel BM. Treatment alternatives for chronic hepatitis B virus infection: a cost-effectiveness analysis. Annals of internal medicine. 2005;142(10):821-31.
28. Wong WW, Lee KM, Singh S, Wells G, Feld JJ, Krahn M. Drug therapies for chronic hepatitis C infection: a cost-effectiveness analysis. Canadian Medical Association Open Access Journal. 2017;5(1):E97-E108.
