# Supplementary material for: Estimating Chronic Hepatitis B Prevalence and Undiagnosed Proportion in Canada, 2007-2021: Mathematical Framework Development
Source: JMIR Public Health Surveill. 2025 Aug 20;11:e66309. doi: 10.2196/66309 (PMC12367349; doi:10.2196/66309)
Supplement: Multimedia Appendix 2 [file publichealth-v11-e66309-s002.docx]

# Appendix 2: Model Dynamics and Likelihood

For each of the model states$-U_{0}$, $D_{0}$, $U_{1}$, ..., $HCC$, $DC$, $LT$, and $PLT$ (see Table 3 below for a full list of model states)—we denote the expected number of individuals in each state in the year $t$ by $U_{0}(t)$, $D_{0}(t)$, ..., and $PLT(t)$. We collect these quantities into a state vector$\vec{x}\left( t \right)= \left[ U_{0}\left( t \right), \ldots, PLT\left( t \right) \right]^{T}$.

We define the state transition matrix, $\boldsymbol{P}$, to be the matrix whose $k, j$th entry, $P_{kj}$ , is the probability of transitioning from the $j$th state to the $k$th state. Finally, we define a source vector, $\vec{s}= {[1-d_{aa}, d_{aa}, 0, ..., 0]}^{T}$ , which controls how new cases of CHB in the year $t$, denoted $u(t)$, are distributed into each of the model states. The evolution of our model is given by:

$$\vec{x}\left( t+1 \right)=\boldsymbol{P}\vec{x}\left( t \right)+\vec{s}u(t)$$

In addition to this evolution equation, at each time $t$, we also calculated four observables, which are used to calibrate the model. The first of these is the number of people receiving treatment, $T_{1}(t)+T_{3}(t)+T_{1}^{C}(t)+T_{3}^{C}(t)$. Based on clinical guidelines [1], treatment is mainly offered to HBeAg+ and HBeAg-CHB patient, and thus our model does not include an inactive treatment state, $T_{2}$. The remaining observables are the number of CHB diagnoses, the number of HCC diagnoses, and the number of late HCC diagnoses, which we define as those diagnosis events that move directly from an undiagnosed state, $U_{i}^{-/C}$, to the $HCC$ state. These are given by fluxes from one set of states to another, and can be calculated as follows:

Let $diag[\vec{x}(t)]$ be the square matrix with $\vec{x}(t)$ along the diagonal and with zeros in all off-diagonal entries. The matrix product $\boldsymbol{P}diag[\vec{x}(t)]$ records the fluxes through the state diagram; its $k,j$th entry represents the number of people moving from the $j$th state to the $k$th state. Denoting the $i$th state by $S_{i}$, we will simplify the notation and let ${(\boldsymbol{P}diag[\vec{x}(t)])}_{S_{k}S_{j}}\equiv{(\boldsymbol{P}diag[\vec{x}(t)])}_{kj}$.

Let $\mathcal{S}$ denote a set of source states, $\mathcal{T}$ a set of target states. Then the expected number of people moving from the source states to the target states at time $t+1$ is given by

$$E\left( \mathcal{S}\to\mathcal{T} \right)\left( t+1 \right)=\sum_{\left( s,\tau\right)\mathcal{\in S\times T}} \left( \boldsymbol{P}diag\left[ \vec{x}\left( t \right) \right] \right)_{\tau s}$$

With this notation in place, we can describe the four observables. The number of new CHB diagnosis events is given by the expected number of people moving from the undiagnosed states, $\mathcal{S=\{}U_{0}, \ldots, U_{3}^{C}\}$, to any of the diagnosed states, plus the number of new cases diagnosed within the first year, $d_{aa}u(t)$. Likewise, the number of new HCC diagnoses is defined as the expected number of people entering the $HCC$ state from any other state, while the number of late HCC diagnoses is defined as the number of people entering the $HCC$ state from an undiagnosed state. Precise formulations are given in Table 2.

Table 2: A description of the model observables. The source and target sets are the undiagnosed states, $\mathcal{S}_{uCHB}=\left\{ U_{0},\ldots,U_{3}^{C} \right\}$, the diagnosed states, $T_{dCHB}=\{D_{0},\ldots,PLT\}$, and the set of all model states,$\mathcal{S}_{all}=\left\{ U_{0},\ldots,PLT \right\}$.

| Observable | Description | Equation |
| --- | --- | --- |
| $O_{Treat}(t)$ | # of patients receiving treatment in year $t$ | $T_{1}(t)+T_{3}(t)+T_{1}^{C}(t)+T_{3}^{C}(t)$ |
| $O_{CHB}(t)$ | # of new CHB diagnoses in year $t$ | $E(\mathcal{S}uCHB\mathcal{\to T}dCHB)(t) + daau(t - 1)$ |
| $O_{HCC}(t)$ | # of new HCC diagnoses in year $t$ | $E(\mathcal{S}_{all} \backslash\{HCC\} \to\{HCC\})(t)$ |
| $O_{LateHCC}(t)$ | # of late HCC diagnoses in year $t$ | $E(\mathcal{S}_{uCHB} \to\{HCC\})(t)$ |

## Likelihood Model

Assuming Gaussian measurement noise, i.e. $Y_{i}(t)=O_{i}(t)+\epsilon_{i}(t)$ with $\epsilon_{i}(t)\sim N(0,\sigma_{i}^{2})$, the log-likelihood of the model parameters, $\phi$, is given by:

$$\mathcal{l}\left( \phi| Y \right)=-\sum_{i\in Ob} \left( \sum_{t=t_{init}}^{t_{fin}} \frac{{(Y_{i}\left( t \right)-O_{i}\left( t \right))}^{2}}{2\sigma_{i}^{2}} \right)$$

where the summation over $i\in Ob$ indicates summation over the four observables:

$Ob = \{Treat, CHB, HCC, LateHCC\}$.

For each observable, we estimate the corresponding variance, $\sigma_{i}^{2}$, by performing linear regression on the associated data, $Y_{i}$, with respect to time and measuring the deviation from the linear trend.

## Model States

Table 3: A list of all model states.

| State | State Description |
| --- | --- |
| $U_{0}$ | Undiagnosed, Immune tolerant |
| $U_{1}$ | Undiagnosed, chronic HBeAg+, without compensated cirrhosis |
| $U_{2}$ | Undiagnosed, inactive, without compensated cirrhosis |
| $U_{3}$ | Undiagnosed, chronic HBeAg-, without compensated cirrhosis |
| $U_{1}^{C}$ | Undiagnosed, chronic HBeAg+, with compensated cirrhosis |
| $U_{2}^{C}$ | Undiagnosed, inactive, with compensated cirrhosis |
| $U_{3}^{C}$ | Undiagnosed, chronic HBeAg-, with compensated cirrhosis |
| $D_{0}$ | Diagnosed but untreated, Immune tolerant |
| $D_{1}$ | Diagnosed but untreated, chronic HBeAg+, without compensated cirrhosis |
| $D_{2}$ | Diagnosed but unterated, inactive, without compensated cirrhosis |
| $D_{3}$ | Diagnosed but untreated, chronic HBeAg-, without compensated cirrhosis |
| $D_{1}^{C}$ | Diagnosed but untreated, chronic HBeAg+, with compensated cirrhosis |
| $D_{2}^{C}$ | Diagnosed but untreated, inactive, with compensated cirrhosis |
| $D_{3}^{C}$ | Diagnosed but untreated, chronic HBeAg-, with compensated cirrhosis |
| $T_{1}$ | Treated, chronic HBeAg+, without compensated cirrhosis |
| $T_{3}$ | Treated, chronic HBeAg-, without compensated cirrhosis |
| $T_{1}^{C}$ | Treated, chronic HBeAg+, with compensated cirrhosis |
| $T_{3}^{C}$ | Treated, chronic HBeAg-, with compensated cirrhosis |
| $HCC$ | Hepatocellular carcinoma |
| $DC$ | Decompensated cirrhosis |
| $LT$ | Liver transplant |
| $PLT$ | Post-liver transplant |

# References

1. Terrault NA, Lok AS, McMahon BJ, Chang KM, Hwang JP, Jonas MM, et al. Update on prevention, diagnosis, and treatment of chronic hepatitis B: AASLD 2018 hepatitis B guidance. Hepatology. 2018;67(4):1560-99.
