# Supplementary material for: Estimating Chronic Hepatitis B Prevalence and Undiagnosed Proportion in Canada, 2007-2021: Mathematical Framework Development
Source: JMIR Public Health Surveill. 2025 Aug 20;11:e66309. doi: 10.2196/66309 (PMC12367349; doi:10.2196/66309)
Supplement: Multimedia Appendix 3 [file publichealth-v11-e66309-s003.docx]

# Appendix 3: Calibration Data

Table 1: CHB diagnosis data, $Y_{CHB}\left( t \right)$

| Year | CHB diagnoses (male) | CHB diagnoses (female) | CHB diagnoses (total) |
| --- | --- | --- | --- |
| 2007 | 3296 | 2792 | 6088 |
| 2008 | 3606 | 2462 | 6068 |
| 2009 | 3302 | 2495 | 5797 |
| 2010 | 3181 | 2362 | 5543 |
| 2011 | 2984 | 2366 | 5350 |
| 2012 | 3061 | 2397 | 5458 |
| 2013 | 3042 | 2447 | 5489 |
| 2014 | 2744 | 2405 | 5149 |
| 2015 | 2735 | 2081 | 4816 |
| 2016 | 2835 | 2155 | 4990 |
| 2017 | 2798 | 2112 | 4910 |
| 2018 | 2673 | 2151 | 4824 |
| 2019 | 2667 | 2127 | 4794 |

Table 2: HCC diagnosis data, $Y_{HCCTotal}(t)$

| Year | HCC diagnoses (male) | HCC diagnoses (female) | HCC diagnoses (total) |
| --- | --- | --- | --- |
| 2007 | 105 | 34 | 139 |
| 2008 | 104 | 37 | 141 |
| 2009 | 118 | 34 | 152 |
| 2010 | 129 | 43 | 172 |
| 2011 | 141 | 46 | 187 |
| 2012 | 152 | 53 | 205 |
| 2013 | 164 | 57 | 221 |
| 2014 | 165 | 57 | 222 |
| 2015 | 181 | 61 | 242 |
| 2016 | 175 | 56 | 231 |
| 2017 | 186 | 57 | 243 |
| 2018 | 181 | 58 | 239 |
| 2019 | 187 | 63 | 250 |

Table 3: Treatment data, $T_{Treat}(t)$

| Year | Treatment (male) | Treatment (female) | Treatment (total) |
| --- | --- | --- | --- |
| 2018 | 12275 | 6737 | 19012 |
| 2019 | 14127 | 8161 | 22288 |
| 2020 | 14100 | 8433 | 22533 |
| 2021 | 14591 | 8721 | 23312 |
